# System Resource Allocation and Rural Industry Revitalization Based on Max-Min Algorithm

**DOI:** 10.1155/2022/7983802

**Published:** 2022-05-31

**Authors:** Qiongyao Dai

**Affiliations:** Law School of Chengdu University, Sichuan, Chengdu 610106, China

## Abstract

At this stage, the research hotspots on wireless multimedia sensor networks mainly include MAC protocol, network security, and node positioning. Research on resource allocation is scarce. Due to the lack of an effective wireless resource allocation strategy, even the most advanced transmission technology will not be able to fully utilize its unique advantages due to unreasonable resource allocation. Therefore, in-depth research on wireless resource allocation strategies is of great significance for the better application of wireless transmission technology. The entire network uses OFDMA mechanism to divide time slots. According to this method, resource allocation can be divided into intercluster and intracluster, and then the Max-Min subcarrier allocation algorithm is innovated and perfected to ensure maximum information transmission speed and throughput and promote information data efficient transmission. Based on the research of wireless sensor system resource allocation technology, China has emphasized industrial prosperity as the key point in its rural revitalization strategy, and doing a good job in technological innovation and industrial innovation is of great significance to economic development. At this stage, the capacity of rural industrial economic development is poor, and the economic level of most rural areas is not high. This situation is inseparable from problems such as limited industrial development and insufficient technological innovation capabilities. In order to promote the prosperity of rural industries and achieve the goal of rural revitalization, we must proceed from the fundamentals and give priority to solving the outstanding problems encountered in the process of rural industrial revitalization. Promoting the revitalization of rural industries can effectively solve the current plight of agricultural development and is an inherent requirement for rural prosperity and the country's prosperity. Aiming at the problems in the process of restricting the revitalization of rural industries, this article adheres to the principle of integrating theory with practice and puts forward practical and feasible measures to solve the problem in a targeted manner.

## 1. Introduction

At this stage, wireless sensor networks are getting more and more attention from people, and they have become one of the current research hotspots. Combining wireless sensors and information-collecting sensors can form a wireless sensor node and multiple wireless sensor nodes. In recent years, a type of wireless sensor network has emerged in the field of wireless sensors [1]. This type of wireless network is mainly responsible for sensor nodes that collect image, video, and audio information from the external environment [2]. It is widely used in the fields of networking, medical treatment, and transportation. In the design process of the wireless sensor network, the idea of OFDMA system resource allocation is introduced into the network, making full use of the advantages of OFDMA system resource allocation multiuser diversity, which can achieve the purpose of quickly integrating information resources, and design according to the characteristics of the network and resource allocation scheme and algorithm that comprehensively consider factors such as throughput, real-time performance, and energy consumption, so that the performance of the entire network can be improved, while meeting the needs of a large amount of real-time data transmission [3, 4]. Therefore, do a good job in the research on sensor networks, and use the system resource allocation of wireless sensors to simulate the rural industry revitalization plan [5]. The whole party's work is focused on the vital issue of rural industry revitalization, which is the lifeblood of the country's prosperity and the stability of the political power, and it is also the foundation of the great revival of the Chinese nation [6]. Since the reform and opening up, even if the income of farmers has increased, the sense of gain and satisfaction of the Chinese people has gradually increased [7]. This is an unprecedented progress in rural development, but the level of rural development still cannot match the level of urban development, and the gap between urban and rural areas is still large [8]. There are many reasons for the gap between urban and rural areas. The main reason is insufficient development and creativity. In order to resolve the main social contradictions, China must attach importance to rural economic development and comprehensively promote the rural revitalization strategy [9]. Agriculture-related industries are the foundation of rural development. In the critical period of building a well-off society in all directions, we will promote the transformation of an agricultural country into an agricultural power. From the perspective of the development of the agricultural industry, solving rural development problems on the basis of agricultural development can effectively promote rural economic development and agricultural modernization, as well as the effective development of rural areas [10]. Comprehensiveness is what has been emphasized in building a moderately prosperous society in an all-round way. This also reveals that the poor and poor rural areas are obstacles to the development of a comprehensive well-off strategy. The solution to poverty alleviation in rural areas is imminent, which requires the revitalization of rural industries.

## 2. Related Work

The literature proposes a brand-new strategy, whose strategy is mainly to achieve the effect of reducing spectrum exchange and improving spectrum utilization through the recognition of radio dynamic spectrum and the use of multiplexing mechanisms. The specific implementation content of this strategy is to use a low-complexity dynamic subcarrier allocation algorithm. Through the two allocation processes of initial allocation and optimized allocation, the number of cycles is greatly reduced, and scientific analysis is carried out according to the actual network characteristics, and perfect resources are designed. Distribution schemes and algorithms then improve the performance of the entire network strategy to ensure real-time and efficient data transmission [11]. The literature proposes an optimization algorithm based on parameter scheduling algorithms that takes into account the actual factors affecting resource allocation. While satisfying real-time business, it can complete the operation of setting the optimization goal to find the maximum throughput [12]. The preliminary design of the optimization model was carried out based on the indicators, and the comprehensive calculation and solution of different target values under a variety of factors were completed, so that different users have basic fairness in the process of use. Taking into account the randomness of the distribution of sensor nodes, the normal progress of the optimization procedure can be ensured by improving the reliability of transmission [13]. After theoretical derivation and demonstration, the maximum benefit theory and convex optimization theory can also be used for resource optimization of wireless sensor networks. The literature shows that, based on in-depth understanding of the overall architecture of wireless sensor networks, combined with its MAC protocol, cluster routing protocol, and multihop network resource allocation strategy, based on the cluster-level topology, multihop wireless sensor networks are actually derived from the rational integration of resources and use, by using the above structure for secondary distribution. Since the network needs to continuously transmit a large amount of real-time information, in order to improve efficiency, the entire network is divided into time slots, and the OFDMA mechanism is used to ensure the real-time and efficient transmission of information. Considering the fairness and complexity of the algorithm in wireless sensor networks and the energy limitation of nodes, an optimized allocation algorithm is proposed. The literature analyzes the four aspects of rural industry integration with agricultural industrial clusters, consolidation of agricultural support and protection, development of characteristics, and consolidation of agricultural support and protection [14]. Among them, it has discovered the current difficulties in the development of industries in rural areas and strives to find a suitable one for China. The path of rural development, the balanced development of urban and rural areas, and the solution of the complex and complex rural industrial revitalization issues and the country's food security issues provide a reliable basis. These experiences are of great reference to the development and promotion of rural areas at home and abroad. The literature states that the prospects for rural development are worrying, and various unbalanced problems have appeared in the development process. Therefore, only by strengthening the development of rural industries can the villages be truly rejuvenated. This often requires the state to take strategic measures to effectively improve poverty and backwardness and solve the root causes of rural poverty. It is not only poverty alleviation, but also rural industries, integration, agricultural industry clusters, development of characteristic industries, strengthening policy support, and increasing technological innovation to improve the industry. At the same time, the support of relevant government departments is also extremely important to ensure the progress of industries and implement rural revitalization strategies. Only in rural areas, the all-round development of the country can further promote agricultural modernization.

## 3. Research on the Basics of Wireless Sensor Systems and Resource Allocation Algorithms

### 3.1. Overview of Resource Allocation in Wireless Sensor Networks

#### 3.1.1. The Concept and Characteristics of Wireless Sensor Networks

Wireless sensor network is a special form of wireless network, which has the basic characteristics and related characteristics of wireless network. Wireless sensor networks not only introduce new features for detecting multimedia information such as images, sounds, and videos, but also usually have microphones and cameras and can also be used for computing and storage communications. Interactive storage and communication from the perspective of perception, capture, etc. can process and transmit audio, video, and digital data and images within your network coverage. This allows the application of wireless multimedia sensor networks to cover various aspects such as smart home; medicine and health; flow monitoring; environmental monitoring and on-site monitoring. The similarities and differences between the two are shown in Table 1.

Therefore, in the process of resource allocation, the algorithm used needs to take into account the influence of external factors that may be encountered in practical applications and avoid multiple restrictions that affect the scientific and reasonableness of the final target value.

#### 3.1.2. The Architecture of Wireless Sensor Network

**Figure 1:**
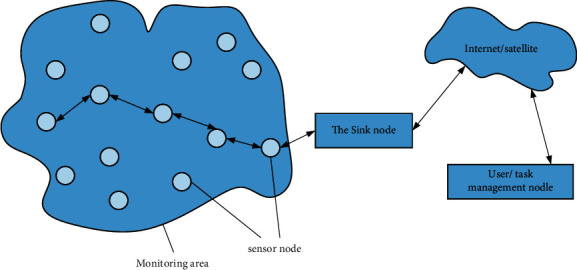
Wireless sensor network architecture.

The common wireless sensor network architecture is shown in Figure 1. The nodes in the monitoring area are randomly distributed in a variety of ways, and they are spontaneous in the process of forming a network. All nodes have the characteristics of switching, interconnection, positioning, and integration. All nodes constitute a micro-embedded system, and this type of system has the problem of poor storage capacity. The hardware device maintains normal operation through battery operation. While processing basic information, the system also manages and integrates data transmitted by other nodes, cooperate with other nodes to perform the specified tasks, and then transmit the data to the sink node. Compared with the above nodes (groups), sink nodes are relatively strong. These nodes (groups) are the direct connection between the sensor network and the external network (such as the Internet), so the collected data is transmitted to the end user.

#### 3.1.3. Sensor Network

**Figure 2:**
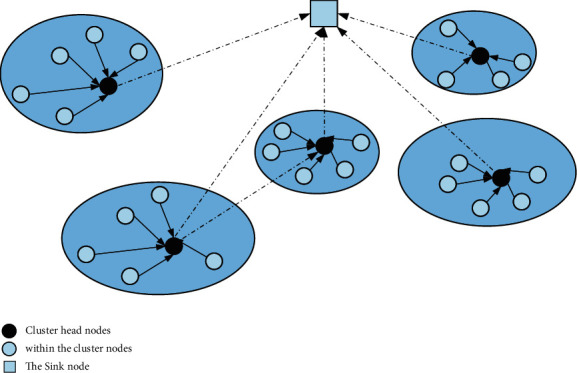
WMSN clustering model.

In the actual application process, it will be required to cover a large area and large-scale sensor network. However, because of the large number of sensor nodes during the operation of the system, it is impossible to achieve long-distance communication in practical applications, which makes the single-layer network structure unscalable, so that the system coverage is larger without reducing the quality of the service area. Generally, a clustered network model is used, as shown in Figure 2.

Through the form of clustering, nodes can be transformed into clusters, which make the system easier to manage after operation and also shorten the communication distance between nodes, which reduces the energy consumption in the process of information transmission between nodes. Enhancing the reliability of network data transmission is an effective way for large-scale wireless sensor networks. The purpose of clustering is to use the limited resources of nodes to complete efficient data collection tasks, thereby saving energy consumption and prolonging the service life of the network.

### 3.2. Resource Allocation in OFDMA System

#### 3.2.1. Wireless Channel Characteristics

Channels can be divided into wired channels and wireless channels according to the transmission medium. Wired channels mainly include cables, optical fibers, and overhead open wires; wireless channels include ultra-short wave and direct microwave propagation. The difference between a wireless channel and a wired channel is that the wireless channel uses electromagnetic waves to transmit information. However, electromagnetic waves are often interfered by various external factors during the transmission process, and the frequency of the receiving end is shifted. This phenomenon is called the Doppler Effect. Taking into account the possible influence of the received signal, the expression of its power is

(1) $$\begin{array}{c} P\left(d\right)={\left|d\right|}^{-n}S\left(d\right)R\left(d\right). \end{array}$$

Equation (1) reflects the three effects of wireless channels on the signal.

The fading formula of the large-scale fading model adopted when the carrier frequency is 2 GHz is

(2) $$\begin{array}{c} L=128.1+37.6\;{\text{log}}_{10}\left(R\right). \end{array}$$

If the path loss and shadow change are combined, the average received power at a distance of *d* from the base station at dozens of wavelength intervals can be expressed as

(3) $$\begin{array}{c} p=p_{0}+10\text{ log}\left(\frac{d}{d_{0}}\right)+X_{\sigma }. \end{array}$$

#### 3.2.2. Principles of OFDMA System Resource Allocation

The RA criterion refers to maximizing the total throughput of non-real-time users through adaptive subcarrier and bit allocation under the constraints of the total transmission power of the system and the user proportional rate. The optimization problem of RA criterion can be modeled as follows:

(4) $$\begin{array}{c} \underset{r_{k,n},p_{k,n}}{\text{max}}\sum_{k=1}^{K}\sum_{n=1}^{N}=\rho_{k,n}r_{k,n}, \end{array}$$

(5) $$\begin{array}{c} \sum_{k=1}^{K}\rho_{k,n}=1, \end{array}$$

(6) $$\begin{array}{c} \sum_{k=1}^{K}\sum_{n=1}^{N}p_{k,n}=P_{\text{total}}. \end{array}$$

The users selected by the maximum C/I scheduling algorithm are

(7) $$\begin{array}{c} k=\underset{i=1_{,-,}K}{\text{arg max}}\left\{{\left(\frac{C}{I}\right)}_{i}\left(n\right)\right\}. \end{array}$$

The priority calculation formula is as follows:

(8) $$\begin{array}{c} P_{k}\left(t\right)=\frac{DRC_{k}\left(t\right)}{R_{k}\left(t\right)}, \end{array}$$

where *DR*  *C*_*k*_(*t*) is the instantaneous data rate. *R*_*k*_(*t*) is the average transmission data rate of user *k*, and the rule can be transformed into

(9) $$\begin{array}{c} R_{k}\left(t\right)=\left\{\begin{array}{c} \left(1-\frac{1}{T_{c}}\right)R_{k}\left(t-1\right)+\frac{1}{T_{c}}DR\;C_{k}\left(t-1\right),\;k=k^{*},\\ \left(1-\frac{1}{T_{c}}\right)R_{k}\left(t-1\right),\;k\neq k^{*}. \end{array}\right. \end{array}$$

Table 2 compares the advantages and disadvantages of the three subcarrier allocation algorithms, the PF algorithm, the Max-Min algorithm, and the MaxC/I algorithm.

### 3.3. Resource Allocation Strategy Based on Clustering and Two-Hop Model

#### 3.3.1. Infinite Sensor Network Model

**Figure 3:**
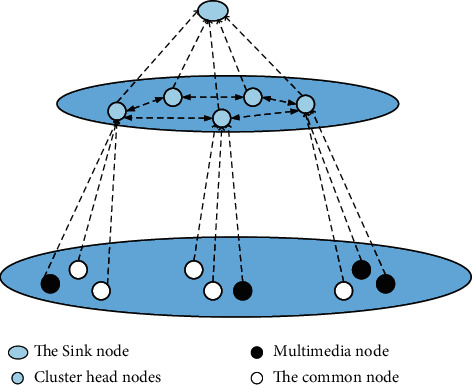
WMSN clustered network model.

The system model is shown in Figure 3. The nodes in the cluster can complete the information transfer between each node and the cluster head node. After the information is transferred to the cluster head node, the cluster head S node starts to transmit information to the sink node. In order to achieve the purpose of simplification of the problem, this article uses a hypothetical way to simulate the cluster head node using a single hop method to transfer the information to the sink node. Sink node has many advantages, and its storage space is extremely powerful. It can transmit the information data received in the process of information transmission to the network outside the system. Through the above methods, it is responsible for the completion of the information transmission between the local network and the external network, communication process.

#### 3.3.2. Optimization Goals and Algorithm Steps

Assuming that the total power of each cluster head node is Pmax, the power *P*_*k*_ allocated by each node in the cluster is calculated according to the equal power allocation on all subcarriers. *N_k_* is the number of subcarriers finally allocated by the kth node, and *P*_*k*_ is the rate of the kth node finally allocated.

(10) $$\begin{array}{c} P_{k}=\frac{P_{\text{max}}N_{k}}{N}. \end{array}$$

Most of the existing energy-saving schemes focus on the design of routing strategies and the reconciliation scheme of cluster head nodes. Considering that extending network life and system energy-saving are eternal themes of wireless sensor network research and design, this chapter mainly discusses resource allocation algorithms. The definition of energy efficiency: the number of successfully transmitted bits per joule of energy (unit: bit/joule). The definition is

(11) $$\begin{array}{c} U\left(R\right)=\frac{R}{P\left(R\right)}\\ =\frac{R}{P_{c}+P_{T}\left(R\right)}. \end{array}$$

In the above formula, *U* (*R*) is called energy efficiency, and *R* is the data transmission rate, which can be expressed by Shannon's formula as

(12) $$\begin{array}{c} R=B\text{log}2\left(1+\frac{P_{T}h}{N_{0}B}\right), \end{array}$$

where *h* is the channel gain, and *N*_0_ is the power spectral density of the noise. *P*_*c*_ is the circuit power, and *P*_*T*_ (*R*) is the transmission power, which can be obtained from the following equation:

(13) $$\begin{array}{c} N_{k}=\overset{N}{\sum }\omega_{k,n}, \end{array}$$

(14) $$\begin{array}{c} P_{T}\left(R\right)=\left(e^{\left(R/B\right)}-1\right)\left(\frac{N_{0}B}{h}\right). \end{array}$$

The goal is to maximize the total throughput. When the sink node allocates resources to each cluster head node, it cannot blindly pursue the maximum throughput, while also ensuring that energy consumption is minimized. As energy consumption is reduced, based on the same energy supply, the requirement of extending the network life cycle can be achieved. Therefore, the optimization goal of the resource allocation algorithm between clusters is to maximize energy efficiency: maxU(R). The target data transfer rate is

(15) $$\begin{array}{c} R^{*}=\underset{R}{\text{arg max}}U\left(R\right)\\ =\underset{R}{\arg\text{ max}}\frac{R}{P_{c}+P_{T}\left(R\right)}. \end{array}$$

Energy efficient resource allocation can be described by an optimization model shown as follows:

(16) $$\begin{array}{c} c^{*}=\underset{c}{\arg\text{ max}}\sum_{i}\frac{R_{i}}{P_{i}\left(R_{i}\right)}\\ =\underset{c}{\arg\text{ max}}\sum_{i}U_{i}\left(R_{i}\right). \end{array}$$

Define *P*_*T*_*i*__(*r*_*i*_) as the transmit power of the sink node on each subcarrier when the node rate is r_*i*, and put *P*_*i*_(*R*_*i*_)=*P*_*c*_+*c*_*i*_*P*_*T*_*i*__(*r*_*i*_) into the formula to get

(17) $$\begin{array}{c} U_{i}\left(R_{i}\right)=\frac{R_{i}}{P_{i}\left(R_{i}\right)}\\ =\frac{c_{i}r_{i}}{P_{c}+c_{i}P_{T_{i}}\left(r_{i}\right)}\\ =\frac{r_{i}}{\left(P_{c}/c_{i}\right)+P_{T_{i}}\left(r_{i}\right)}. \end{array}$$

Let *V*_*i*_(*c*_*i*_)=(*r*_*i*_/(*P*_*c*_/*c*_*i*_)+*P*_*T*_*i*__(*r*_*i*_)), then formula (16) is equivalent to

(18) $$\begin{array}{c} c^{*}=\underset{c}{\text{arg max}}\sum_{i}V_{i}\left(c_{i}\right). \end{array}$$

Because *V*_*i*_(*c*_*i*_) is strictly concave in function image analysis, equation (18) can calculate the optimal solution. The Lagrangian function that can be constructed through the above rules and formulas is

(19) $$\begin{array}{c} L\left(c,\lambda \right)=\sum_{i=1}^{N}V_{i}\left(c_{i}\right)-\lambda \left(\sum_{i=1}^{N}c_{i}-K\right). \end{array}$$

Let (*∂L*/*∂c*_*i*_)=*V*_*i*_′(*c*_*i*_) − *λ*=0, then we can derive

(20) $$\begin{array}{c} c_{i}=\frac{\sqrt{\left(P_{c}r_{i}/\lambda \right)}-P_{c}}{P_{T_{i}}\left(r_{i}\right)}. \end{array}$$

From equation (18), *λ* can be obtained, and then the optimal subcarrier allocation for node *i* can be obtained as

(21) $$\begin{array}{c} c_{i}^{*}=V_{i}^{\prime }-1\left(\lambda^{*}\right), \end{array}$$

where *λ*^*∗*^ is determined by the following formula:

(22) $$\begin{array}{c} \sum_{i}c_{i}^{*}=\sum_{i}V_{i}^{\prime 1}\left(\lambda^{*}\right)\\ =K. \end{array}$$

For each node, calculate the number of initial available subcarriers:

(23) $$\begin{array}{c} m_{k}=\frac{R_{\text{min}}^{k}}{R_{\text{max}}},\;k=1,\ldots ,K. \end{array}$$

When the total number of allocated subcarriers is less than N, for each node,

(24) $$\begin{array}{c} G_{k}=\frac{m_{k}+1}{H_{k}}f\left(\frac{R_{\text{min}}^{k}}{\left(m_{k}+1\right)}\right)-\frac{m_{k}}{H_{k}}f\left(\frac{R_{\text{min}}^{k}}{m_{k}}\right),\;k=1,\ldots ,K. \end{array}$$

### 3.4. Performance Simulation and Analysis

#### 3.4.1. Simulation Environment and Parameter Settings

In the process of simulation, it is necessary to select a highly generalized, rigorous, scientific, reasonable, and maneuverable implementation plan, carry out the preliminary establishment of the system model, and conduct a variety of factors that may affect the policy process and results. Through the analysis, the performance of the network system is comprehensively evaluated by using a variety of basic and other parameters. Simulation related parameter settings are shown in Table 3.

**Figure 4:**
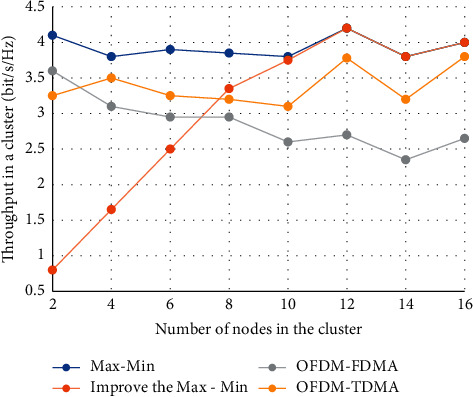
Throughput comparison within a cluster.

Figure 4 is the variation curve of the intracluster throughput with the number of intracluster nodes in different resource allocation algorithms.

**Figure 5:**
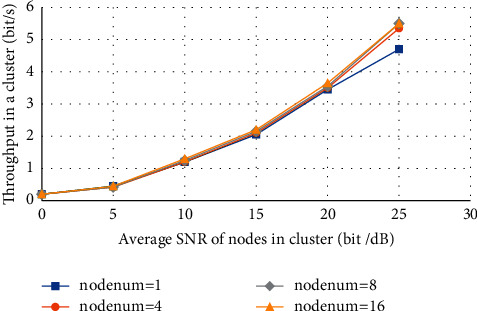
The variation curve of throughput in a cluster with the average SNR of the node.

As shown in Figure 5, the Max-Min algorithm and the Max-Min optimization algorithm used in this paper have the same characteristic attributes in terms of system capacity. Both their system capacities show an improved state when the number of nodes in the network increases. On the contrary, the total system capacity of the OFDM-FDMA algorithm and the OFDM-TDMA algorithm shows a decreasing trend when the number of nodes increases.

Figure 5 shows the variation curve of the throughput within the cluster with the average SNR of the node under the condition of different numbers of nodes.

**Figure 6:**
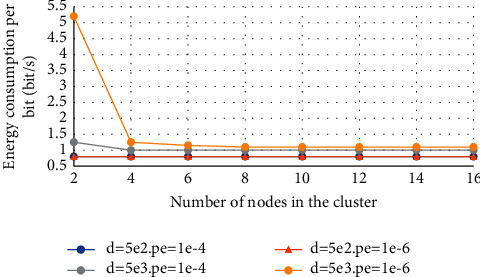
The energy consumption under different system parameters varies with the number of nodes in the cluster.

It can be seen from Figure 6 that, with the continuous increase of node SNR, the total throughput within the cluster continues to increase, and the throughput within the cluster increases with the increase of the number of nodes.

Figure 6 is the variation curve of the energy consumption E_bt_ (that is, energy efficiency) per bit of information transmitted with the number of nodes in the cluster N under different transmission distances and bit error rate (BER) requirements.

The number of nodes in the cluster varies from 2 to 16 with an interval of 2. In the figure, *d* represents the distance from the node in the cluster to the cluster head node, and Pe is the BER requirement of each node. The calculation formula of *E_bt_* is as follows:

(25) $$\begin{array}{c} E_{bt}=\left(1-\alpha \right)\frac{N}{4}N_{0}{\left[\frac{\left(2N-1/N\right)}{p_{e}}\right]}^{\left(1/N\right)}\frac{\left(4\pi d^{2}M_{l}N_{f}\right)}{G_{t}G_{r}\lambda^{2}\rho }+\frac{P_{c}}{R_{b}}. \end{array}$$

The value of each parameter in formula (25) is shown in Table 4.

**Figure 7:**
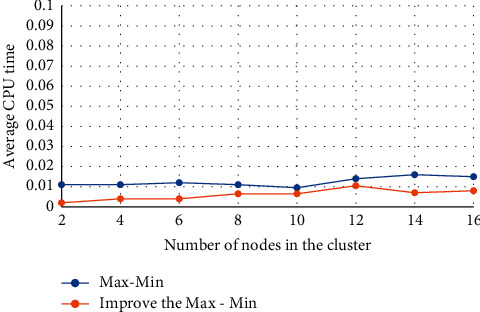
Comparison of the average CPU time of the algorithm.

Figure 7 shows the comparison between the processing time of the Max-Min algorithm and the time used by its optimization algorithm during the simulation test. It can be understood that the processing time of the optimized algorithm is less than that of the original algorithm, and at the same time, the amount of calculation in the simulation process is smaller. Therefore, it can be seen that the calculation speed of the optimized algorithm is better.

## 4. Research on the Issue of Industrial Revitalization under the Background of Rural Revitalization Strategy

### 4.1. Overview of Rural Revitalization Strategy

In the new historical position of achieving a well-off society, building a modern and powerful socialist country in an all-round way, and making the Chinese nation prosperous, agriculture should be the top priority, focusing on rural areas and peasants that are related to national livelihoods in the new era, and improving the urban integration system. Speed up the modernization of rural areas. The first is to implement the rural revitalization strategy, promote the modernization of the agricultural economy, realize the overall development of the rural agricultural industry, and promote the coordinated social development of rural social management and rural income. The countryside is an organism, and all aspects must be a whole. The second is to promote the integration of secondary and tertiary industries. By cultivating new agricultural units, improving the comprehensive security system, breaking through element constraints, improving the tertiary industry benefit link mechanism, changing the situation of closed development of traditional agriculture, and gradually extending all links of agricultural development to rural, suburban, and even rural areas, promote the integration of urban and rural areas. The flow of time contributes to the optimal allocation of resources. The third is to adhere to the priority of agricultural and rural development, focus on the “three rural” issues, increase poverty alleviation, solve outstanding problems in the development of rural industries, focus on grain production, optimize the industrial structure through technological innovation, and inject new vitality into rural revitalization. The fourth is to comprehensively deepen rural reforms and improve the rural governance system. It is necessary to strengthen the construction of the basic land management system, the land circulation system, and the land collective property rights system to provide a solid organizational guarantee for rural development and marketing.

It is necessary to solve the problem of the poor in rural areas and achieve the overall victory of poverty alleviation in rural areas. We must rely on industrial revitalization to improve the quality of farmers. With income, the countryside will become rich, and the countryside will prosper in an all-round way. At present, most rural areas still rely on agriculture as their main source of income. Therefore, to build a prosperous society in multiple ways, the first task is to believe that agriculture will revitalize the landscape. It is necessary to develop characteristic industries in rural areas, increase employment opportunities and positions for farmers, give farmers opportunities to show their skills, improve the living standards of the poor, and promote the sustainable and healthy development of rural industries.

### 4.2. The Low Level of Industrial Integration Development Makes It Difficult to Form a Coordinated Development Situation

The integration of primary, secondary, and tertiary industries in rural areas is a new type of intensive agriculture model. With agriculture as the cornerstone, resources, capital, technology, management, and other elements are gathered to form an agricultural production chain. The main approach is to innovate systems and mechanisms and the linkage of interests, and to transform the mode of agricultural development to build an integrated, coordinated, and enriched modern agricultural system to form an integrated development pattern of grassroots, grassroots, and grassroots levels. The development of primary, secondary, and tertiary industries in rural areas has practical significance. In the context of the traditional model, production is the main activity of farmers. In an integrated environment, the focus has begun to shift from production to operation, which places demands on the main operators of rural industries. Therefore, the main agricultural operators must not only have certain management capabilities, but also have the ability to obtain information, market awareness, and financing skills. At the same time, many problems that need to be resolved were discovered in the course of business operations.

At this stage, capital, land, and technology are restricting the integrated development of China's rural primary, secondary, and tertiary industries to varying degrees. From the current development practice, it can be seen that factors such as the serious shortage of talents for the integrated development of rural primary, secondary, and tertiary industries have restricted the development of rural industries in China to a certain extent. There is a serious shortage of rural industrial land. In the actual process of the integrated development of the primary, secondary, and tertiary industries in rural areas, industrial land rarely has a stable supply. Unlike traditional agriculture, the integration of the three major industries in rural areas has greater demand for land. With the gradual increase in the added value of products, the types of products are rich and diverse. Therefore, in rural areas, the land used by the three major industries is in great demand. Therefore, in the process of integrated development of primary, secondary, and tertiary industries in rural areas, a larger-scale infrastructure production area is needed. For example, the development of the “Nongjiale” project not only needs to protect the land where crops are grown, but also needs to be equipped with various land sites such as reception land, residential land, catering land, and parking lot. Most of the catering land for integrated projects is directly open-air. In addition, it is temporarily mixed with parking lot land; the development of agroprocessing industry also requires a large amount of land for storage management and operation. At the same time, when applying for land use indicators in different regions, problems such as long time-consuming, high cost, high difficulty, and few indicators were discovered. Due to the inefficient and inefficient rural land, the problem of insufficient land supply has become the biggest obstacle to the integrated development of the rural tertiary industry. In addition, in the process of implementing rural land transfer, many problems have been discovered. For example, the mechanism is loose and imperfect. The traditional concept of most farmers cannot be shaken, and some farmers do not want to transfer their land to others because they are afraid of losing money. Insufficient understanding of land transfer policies has led to too low participation in land transfer, which has seriously affected the insufficient supply of rural industrial land.

### 4.3. Main Measures to Promote the Revitalization of Rural Industries

The coordinated development of various aspects of agricultural industrial activities enables China's agricultural production practices to maintain a stable and balanced development. The integrated development of rural industries requires a large agricultural production scale, strong economic reality, and strong management capabilities, organized and guided by a new type of agricultural business entity, gathering relatively small-scale individual farmers, and transforming from an operation mode that farmers directly participate in to a new type of agricultural business entity that participates indirectly or directly. The new type of agricultural business is essentially different from the traditional agricultural business. The establishment of a new type of rural industry is of great significance for ensuring the healthy development of China's agriculture.

In terms of promoting the integrated development of rural primary, secondary, and tertiary industries, because the linkage mechanism in terms of interests is particularly weak, this has led to the stagnation of the process of agricultural industrialization. How to protect the interests of multiple parties has become the primary issue in rural development. The process of modern agricultural industrialization: the key to solving this problem is to build a reasonable interest linkage mechanism, so that the interests of farmers and leading enterprises can be distributed reasonably, and the actual contradiction between the big market and small farmers can be effectively resolved. The specific policies include the following: first, the establishment of a risk prevention and protection system. Agricultural production is very susceptible to fluctuations due to natural conditions. Active agricultural insurance and agricultural purchase protection prices (as far as possible to ensure higher than market returns) can minimize market price fluctuations and losses caused by natural disasters and continuously improve farmers and enterprise's risk prevention awareness and ability.

Establish authoritative industry associations to protect social services. At present, the number of agricultural service organizations is still very small, and the quality is quite different. This makes the establishment of authoritative industry associations to optimize and upgrade agricultural industrial clusters imminent. An authoritative industry association is an organization that provides professional services. Its role is to be responsible for industrial operation, economic operation, promotion, and marketing, closely contact the government and farmers, farmers, and markets, and provide market information guidance and technical promotion services, as well as agricultural training and education. At the same time, it supervises the internal entities of the cluster, so that it can quickly and effectively solve the problems of backward technology, single mode of production, and untimely access to market information in the development of rural industries. In addition, industrial clusters exist in various forms such as agricultural product industrial clusters, agricultural cooperative economic clusters, and intermediary clusters. Trade associations are based on the premise of formulating rules and regulations or standards that meet the requirements of industrial development, so as to supervise and manage the main bodies of agricultural industrial clusters and promote the development of agricultural industrialization, marketization, and large-scale development. This makes the industry associations highly sought after by developed countries. However, it is insignificant that the development of agricultural industrialization only depends on the strength of individual farmers. Therefore, while establishing agricultural associations and other service organizations, it is also extremely important to give play to the guiding and coordinating role of the government. By vigorously supporting agricultural development, we encourage the introduction of advanced foreign countries, that is, the use of agricultural information network to realize the sharing of information and data resources and realize the win-win results of increasing farmers' income and effective government management.

## 5. Conclusion

Wireless sensors belong to a practical application of wireless sensor networks. What type of resource allocation scheme is used to meet the energy-constrained requirements and improve the overall performance of the wireless multimedia human sensor network has become the key to this paper. Therefore, a resource allocation scheme that comprehensively considers energy consumption, throughput, and fairness is a huge demand for wireless multimedia sensor networks. This paper also proposes a resource allocation scheme in wireless multimedia sensor networks: based on cluster hierarchical topology, using the multihop nature of secondary resource allocation. Seeking to maximize the throughput in the cluster to ensure the real-time and efficient transmission of information, a subcarrier allocation algorithm based on energy-saving ideas is designed between clusters. When the sink node allocates subcarriers to each cluster head node; it takes energy efficiency maximization as the optimization goal to maximize the energy efficiency of the entire network. The revitalization of rural industries is currently a key issue that needs to be resolved and adjusted. Only when rural industries are revitalized can the gap between the rich and the poor be reduced in a true sense. However, in the implementation of rural industries in different regions, there are still many problems that need to be resolved, and the difficulties in the implementation process still need to be overcome. Through research, investigation, and document review and comparison, in the application of various theories, this paper comparatively analyzes the current situation of rural industry development and the existing problems and discusses the corresponding solutions to the revitalization of rural industries.

## Figures and Tables

**Table 1:** Similarities and differences between WSN and WMSN.

		WSNs	WMSNs
Same point	Self-organization, multi-hop routing, resource-constrained, energy-sensitive, unattended, etc.		

Difference	Energy consumption distribution	Low energy consumption, mainly concentrated on infinite techniques, and the energy consumption distribution is “aggregated”	The energy consumption is high, and the energy consumption of wireless transmission and reception is basically the same as that of data collection and processing, and it is evenly distributed.
	Processing task	Simpler, simple addition, subtraction, multiplication, division, averaging, data aggregation, etc.	Complex, image compression coding, distributed video processing, information fusion, etc.
	QoS requirements	Low requirements, sacrifice QoS in exchange for minimizing energy consumption	Strong real-time. Higher system throughput can adapt to different application requirements
	Function application	Simple function, limited amount of perceptual information, simple environment monitoring and other occasions	Abundant perceptual media, fine-grained and precise environmental information monitoring, capable of completing complex tasks such as target detection, recognition, positioning, and tracking
	Sensing model	Omnidirectional, can perceive data from any direction	Most have strong directionality
	The core issue	Minimize energy consumption	Pursue the minimization of energy consumption while meeting QoS

**Table 2:** Advantages and disadvantages of OFDMA system subcarrier allocation algorithm.

Algorithm name	Advantage	Disadvantage
Maximum carrier-to-interference ratio algorithm	Best single user, maximum throughput	The only goal is to maximize the total system throughput, which does not meet the fairness criterion

Minimal capacity user maximization algorithm	Ensure the fairness of throughput between users, and the algorithm complexity is low	Does not consider the QoS requirements of different business users

Proportional fairness algorithm	Different trade-offs can be made between throughput and fairness	In the case of multiple services, the number of remaining subcarriers is greater than the number of users

**Table 3:** Simulation parameter settings.

Number of clusters	7
Sink node maximum transmit power	33 dBm
Total system bandwidth	1 MHz
Number of subcarriers to be allocated	128
Noise power spectral density	–80 dBm/Hz
Mean shadow fading	0
Path loss	128.4 + 37.9log10(d)
Path loss index	4
Shadow fading variance	11 dB
Shadow fading standard deviation	8 dB
System BER requirements	10^−3^

**Table 4:** System parameter values.

*f*_*c*_=2.5*GHz*	*η*=0.36
*G*_*r*_ *G*_*t*_=5*dBi*	*M*_*L*_40 *dB*
*B*=10*KHz*	*P*_*mix*_=30.5*mW*
*P*_*syn*_=50.0*mW*	*P*_*LNA*_=20.0*mW*
*P*_*filt*_=*P*_*filr*_=2.6*mW*	*N*_*f*_=10*dB*

## Data Availability

The data used to support the findings of this study are available from the corresponding author upon request.
